# Supplementary figures and images for: The interplay of Rac1 activity, ubiquitination and GDI binding and its consequences for endothelial cell spreading
Source: PLoS One. 2021 Jul 12;16(7):e0254386. doi: 10.1371/journal.pone.0254386 (PMC8274835; doi:10.1371/journal.pone.0254386)

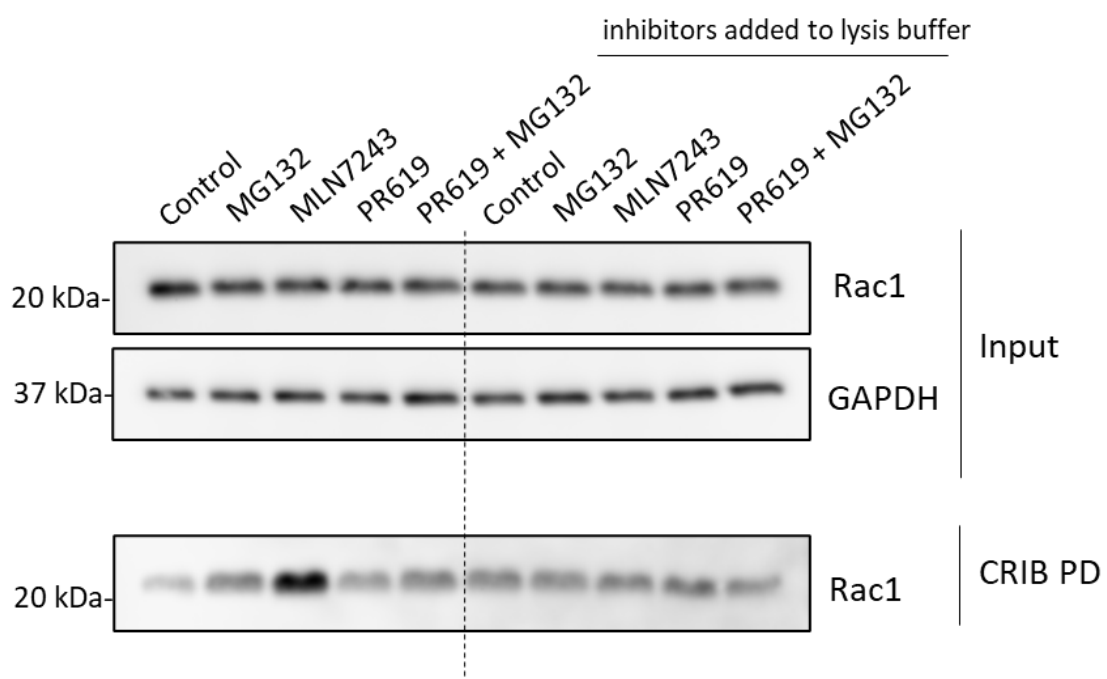

Supplement: S1 Fig — HUVECs were treated with indicated inhibitors for two hours before performing CRIB pulldown, or inhibitors were added in the lysis buffer only. (PDF) [file pone.0254386.s001.pdf]

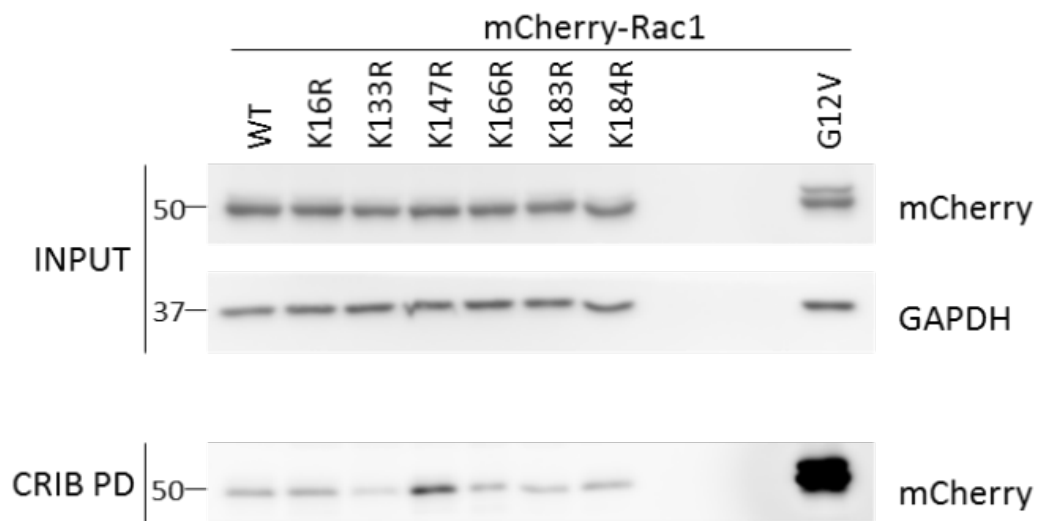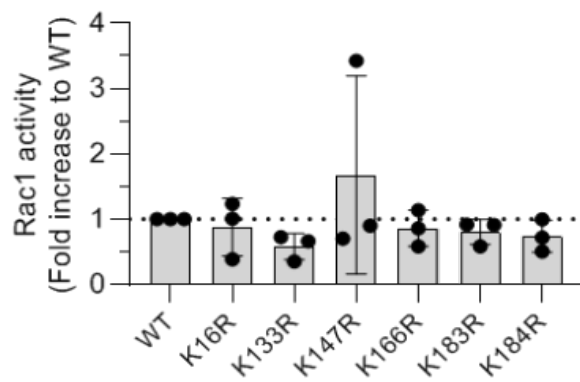

Supplement: S3 Fig — mCherry-Rac1 and mutants thereof were ectopically expressed in HEK293T cells and CRIB pulldown was performed as described in the materials and methods. Representative Western Blot and analysis of n = 3 independent experiments. (PDF) [file pone.0254386.s003.pdf]

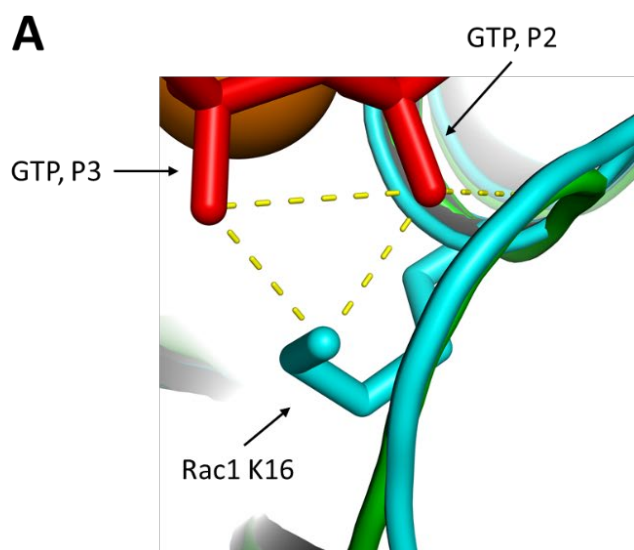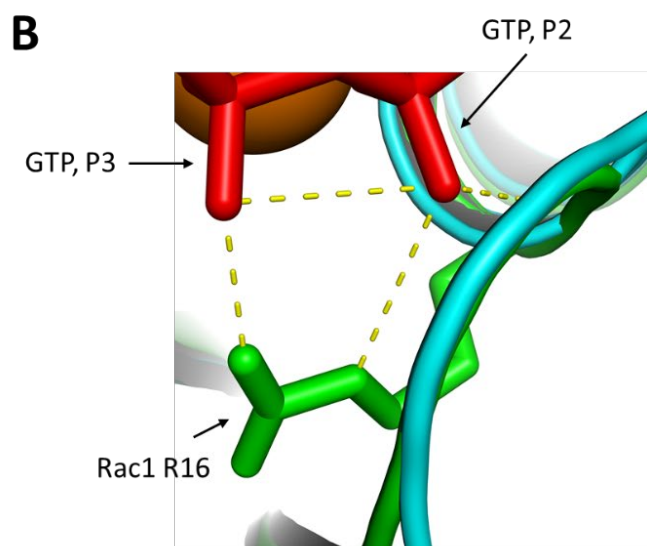

Supplement: S4 Fig — Predicted interaction of (A) Rac1 K16 and (B) Rac1 R16 showing differential interaction with the GTP molecule. Pymol software was used for the protein structure alignment of the GNP-loaded WT and K16R Rac1. The structure of the wild type Rac1 was obtained from Protein Research Database (ID: 3th5) and the structure of the K16R mutant was obtained by modelling using the Phyre2 server (The Phyre2 web portal for protein modeling, prediction and analysis; [44]). (PDF) [file pone.0254386.s004.pdf]
