# Supplementary material for: The interplay of Rac1 activity, ubiquitination and GDI binding and its consequences for endothelial cell spreading
Source: PLoS One. 2021 Jul 12;16(7):e0254386. doi: 10.1371/journal.pone.0254386 (PMC8274835; doi:10.1371/journal.pone.0254386)
Supplement: S2 Fig — (PDF) [file pone.0254386.s002.pdf]

```
H.sapiens         1  MQAIKCVVVGDGAVGKTCLLISYTTNAFPGEYIPTVFDNYSANVMVDGKPVNLGLWDTAG
P.troglodytes     1  MQAIKCVVVGDGAVGKTCLLISYTTNAFPGEYIPTVFDNYSANVMVDGKPVNLGLWDTAG
M.mulatta         1  MQAIKCVVVGDGAVGKTCLLISYTTNAFPGEYIPTVFDNYSANVMVDGKPVNLGLWDTAG
B.taurus          1  MQAIKCVVVGDGAVGKTCLLISYTTNAFPGEYIPTVFDNYSANVMVDGKPVNLGLWDTAG
M.musculus        1  MQAIKCVVVGDGAVGKTCLLISYTTNAFPGEYIPTVFDNYSANVMVDGKPVNLGLWDTAG
D.rerio           1  MQAIKCVVVGDGAVGKTCLLISYTTNAFPGEYIPTVFDNYSANVMVDGKPVNLGLWDTAG
D.melanogaster    1  MQAIKCVVVGDGAVGKTCLLISYTTNAFPGEYIPTVFDNYSANVMVDAKPINLGLWDTAG
                     ***********************************************.**.*********
                                    K16

H.sapiens        61  QEDYDRLRPLSYPQTDVFLICFSLVSPASFENVRAKWYPEVRHHCPNTPIILVGTKLDLR
P.troglodytes    61  QEDYDRLRPLSYPQTDVFLICFSLVSPASFENVRAKWYPEVRHHCPNTPIILVGTKLDLR
M.mulatta        61  QEDYDRLRPLSYPQTDVFLICFSLVSPASFENVRAKWYPEVRHHCPNTPIILVGTKLDLR
B.taurus         61  QEDYDRLRPLSYPQTDVFLICFSLVSPASFENVRAKWYPEVRHHCPNTPIILVGTKLDLR
M.musculus       61  QEDYDRLRPLSYPQTDVFLICFSLVSPASFENVRAKWYPEVRHHCPNTPIILVGTKLDLR
D.rerio          61  QEDYDRLRPLSYPQTDVFLICFSLVSPASFENVRAKWYPEVRHHCPNTPIILVGTKLDLR
D.melanogaster   61  QEDYDRLRPLSYPQTDVFLICFSLVNPASFENVRAKWYPEVRHHCPSTPIILVGTKLDLR
                     *************************.********************.*************

H.sapiens       121  DDKDTIEKLKEKKLTPITYPQGLAMAKEIGAVKYLECSALTQRGLKTVFDEAIRAVLCPP
P.troglodytes   121  DDKDTIEKLKEKKLTPITYPQGLAMAKEIGAVKYLECSALTQRGLKTVFDEAIRAVLCPP
M.mulatta       121  DDKDTIEKLKEKKLTPITYPQGLAMAKEIGAVKYLECSALTQRGLKTVFDEAIRAVLCPP
B.taurus        121  DDKDTIEKLKEKKLTPITYPQGLAMAKEIGAVKYLECSALTQRGLKTVFDEAIRAVLCPP
M.musculus      121  DDKDTIEKLKEKKLTPITYPQGLAMAKEIGAVKYLECSALTQRGLKTVFDEAIRAVLCPP
D.rerio         121  DDKDTIEKLKEKKLTPITYPQGLAMAKEIGAVKYLECSALTQRGLKTVFDEAIRAVLCPP
D.melanogaster  121  DDKNTIEKLRDKKLAPITYPQGLAMAKEIGAVKYLECSALTQKGLKTVFDEAIRSVLCPV
                     ***.*****..***.***************************.***********.****.
                                 K133          K147                K166

H.sapiens       181  PVKKRKRKCLLL
P.troglodytes   181  PVKKRKRKCLLL
M.mulatta       181  PVKKRKRKCLLL
B.taurus        181  PVKKRKRKCLLL
M.musculus      181  PVKKRKRKCLLL
D.rerio         181  PVKRRRRRCLLL
D.melanogaster  181  LQPKSKRKCALL
                     ..[illegible]..*.*.**
                     K183
                      K184
```
