# Supplementary material for: The interplay of Rac1 activity, ubiquitination and GDI binding and its consequences for endothelial cell spreading
Source: PLoS One. 2021 Jul 12;16(7):e0254386. doi: 10.1371/journal.pone.0254386 (PMC8274835; doi:10.1371/journal.pone.0254386)

Supplemental information: full blots

All images were obtained using the AI600 chemiluminescent imager (GE Healthcare). Headings above the figures correspond to the figure panels these blots were used for. Images made including the marker are by default auto-contrasted. Images without marker represent the actual signal and were used for the manuscript figures.

Full blots for Figure 1A

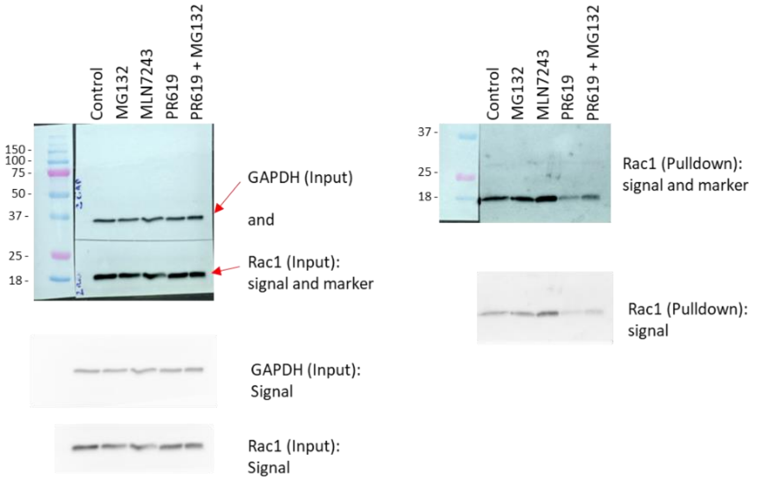

Full blots for Figure 2C

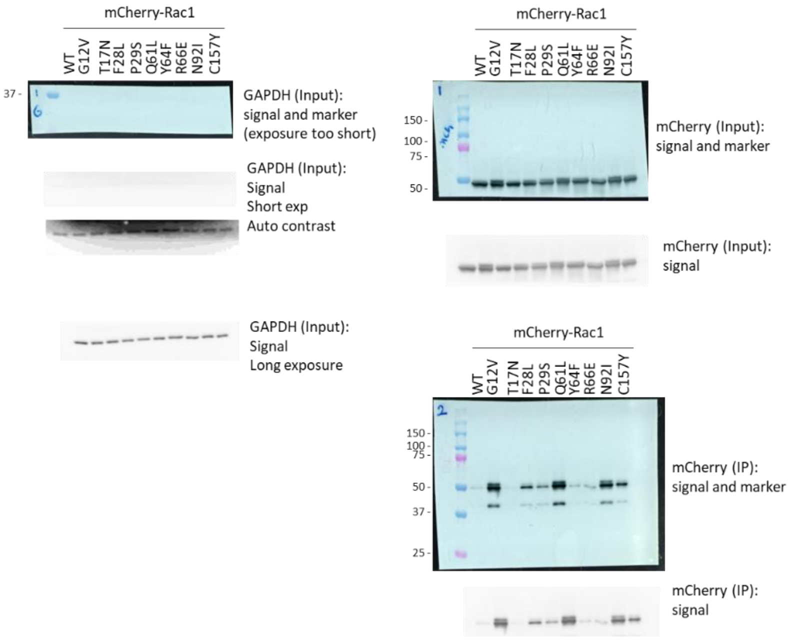

Full blots for Figure 2D

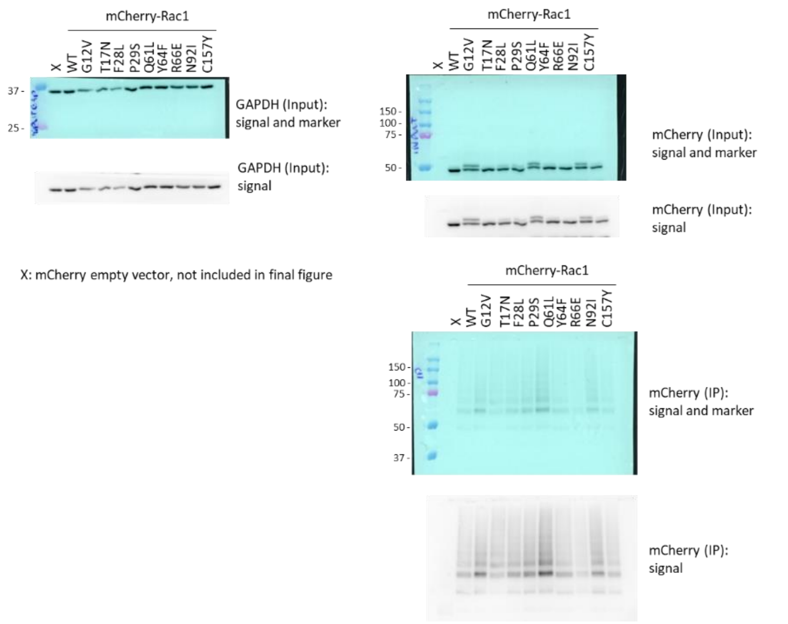

Full blots for Figure 3A

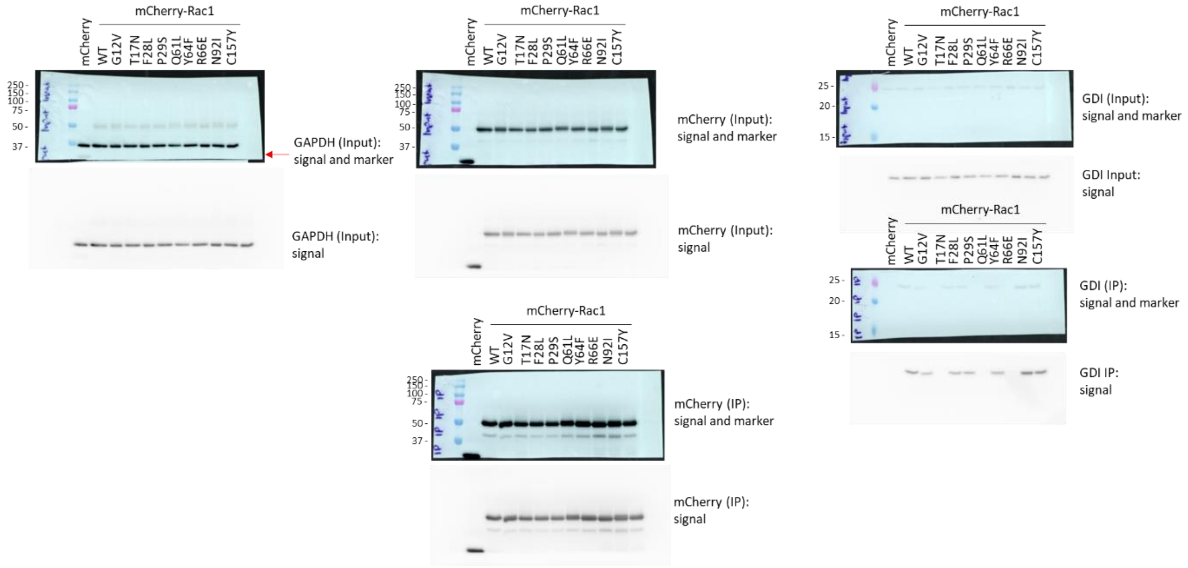

Full blots for Figure 5C

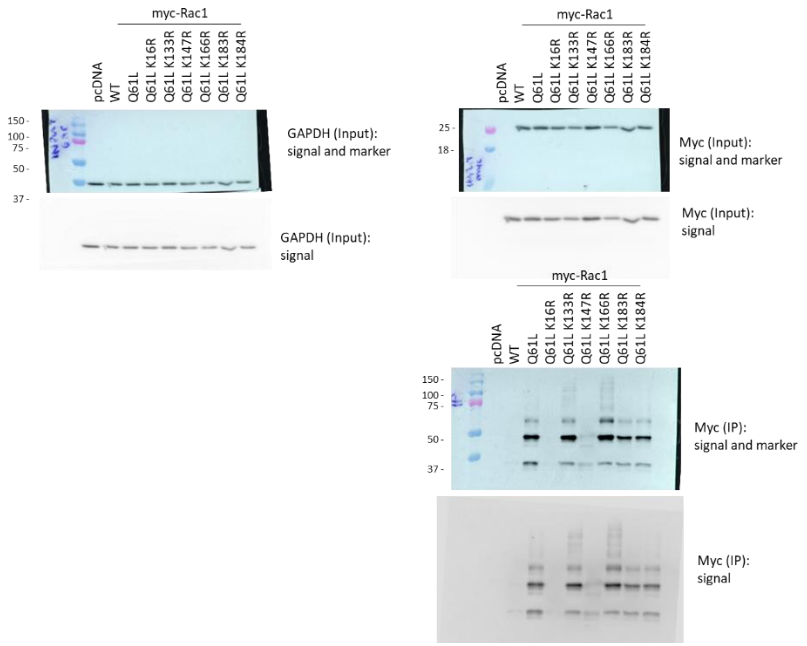

Full blots for Figure 5D

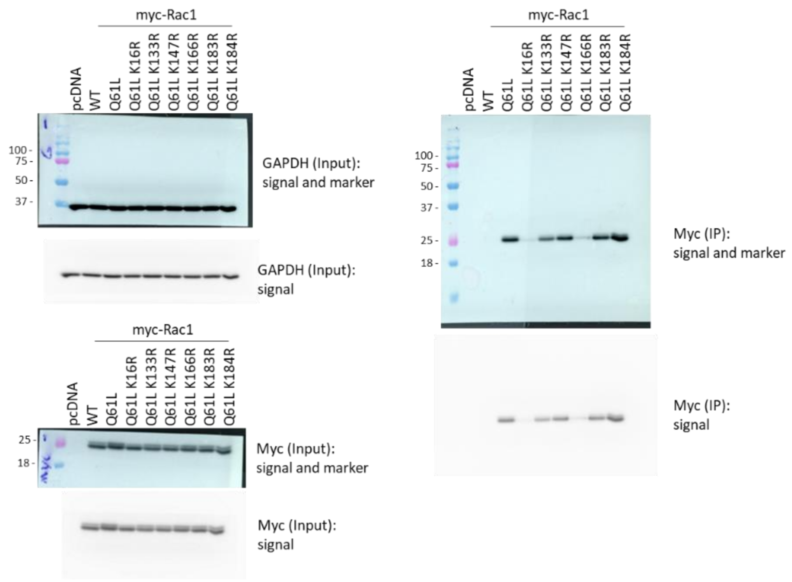

Full blots for Figure 5E

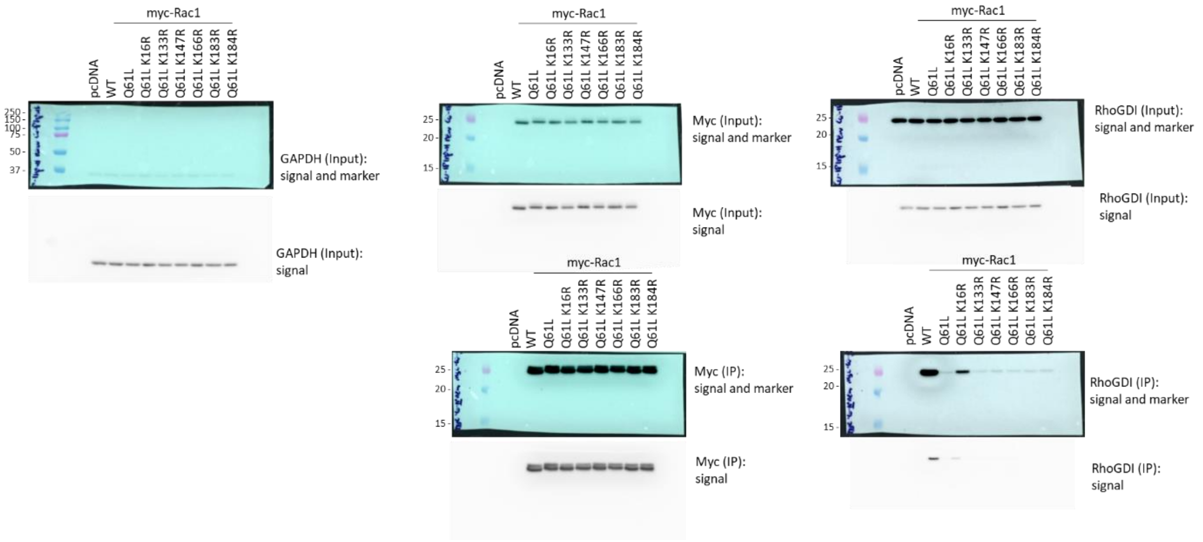

Full blots for Supplemental Figure 1

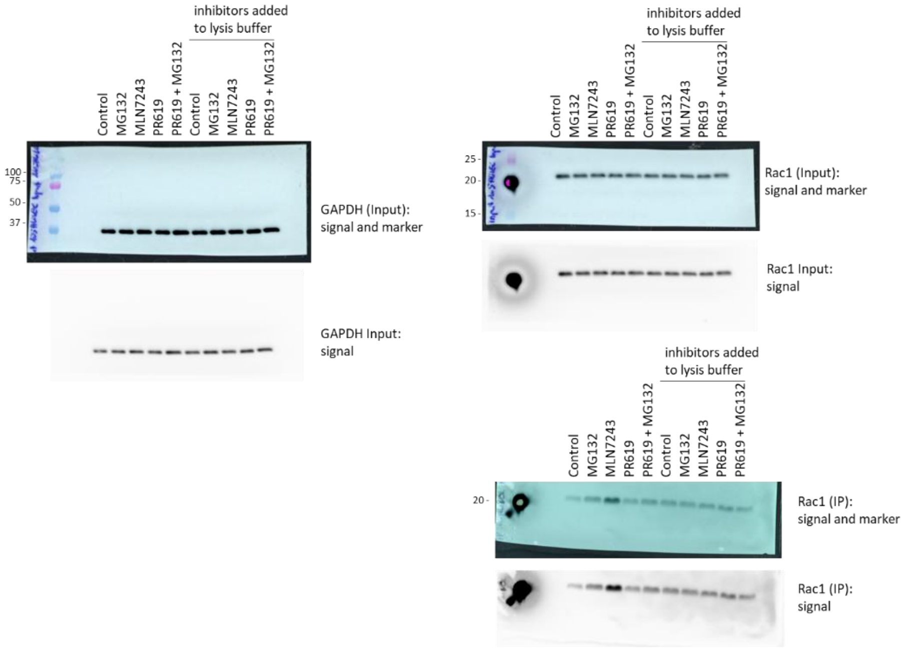

Full blots for Supplemental Figure 3

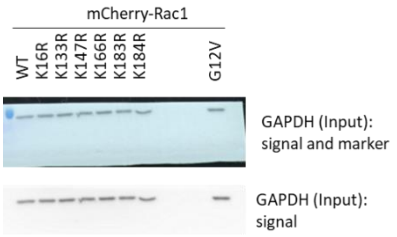

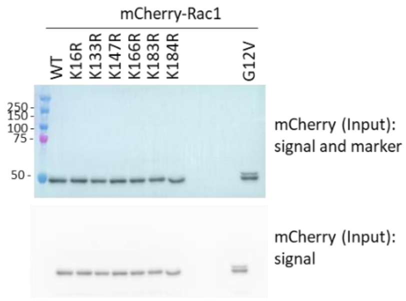

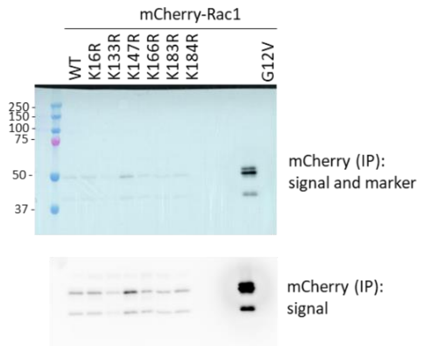

Supplement: S1 Raw images — (PDF) [file pone.0254386.s005.pdf]
