# Supplementary material for: The interplay of Rac1 activity, ubiquitination and GDI binding and its consequences for endothelial cell spreading
Source: PLoS One. 2021 Jul 12;16(7):e0254386. doi: 10.1371/journal.pone.0254386 (PMC8274835; doi:10.1371/journal.pone.0254386)
Supplement: Attachment — Submitted filename: Response to Reviewers.docx [file pone.0254386.s006.docx]

**Response to reviewer’s comments**

Reviewer #1: In this manuscript, Majolée et al. explore relationships between Rac1 activity, GDI binding and ubiquitination. There is a lot of good data of interest to the cell biology community in this manuscript around the effects of several Rac1 mutants on Rac1 localization and function in cell lines. However, the overall conclusions in terms of effects on Rac1 ubiquitinylation, GDI interactions and activity are not convincing or complete, as detailed below.

Major

1. In Fig. 1, the authors incubate HUVECs for 2 h with ubiquitin and proteasome associated inhibitors and then follow Rac1 activity by PAK-CRIB pull-down and Rac1 Western blot. The authors conclude that there is some role of for ubiquitin and proteasome in regulating Rac1 activity. The results are interesting, but slightly difficult to interpret without the inclusion of any controls. For example, it is not clear if the compounds actually affect Rac1 activity, or simply an interaction between Rac1 and PAK. Does the compound have any effect on CRIB pulldown of pure GDP or GTPγS-loaded Rac1?

*The interaction between Rac1 and PAK is the most widely used measure of Rac1 activity in cells, which is why we chose this method for measurement of Rac1 activity. In order to address this reviewers question if the compounds affect the interaction between Rac1 and PAK directly, rather than affecting the activity of Rac1, we added the compounds directly to the lysis buffer when performing the CRIB pulldown (Supplemental Figure 1 and see below). In parallel, cells were treated with the compounds for two hours before performing the pulldown, as was done before in Figure 1 (here left side of the panel). In these experiments, we have not observed any clear effect of MG132, MLN7243, PR619 or the combination of MG312 and PR619 on Rac1 interaction with CRIB peptide when the compounds were added to the lysis buffer directly. This result shows that the compounds do not interfere with the interaction between the PAK CRIB domain and activated Rac1. Thus, we conclude that the effect of the E1 inhibitor MLN7243, resulting in increased Rac1 binding to the CRIB peptide , is due to its consequences for cellular signaling pathways that control Rac1 (in)activation.*

*As the reviewer suggested, we also performed the CRIB pulldown from purified Rac1 in presence of GDP or GTPγS. Here, we do not observe any difference in CRIB binding between different conditions, showing that the direct interaction between Rac1 and PAK is not affected.*

2. It is not clear whether HUVECs in their basal state are most appropriate for these kinds of studies – or if some stimulated state that activates Rac1 would be more informative (i.e., thrombin stimulation).

*We decided to introduce the spectrum of activating mutations in Rac1, which are diverse in terms of activation mechanism and their effect on Rac1 signaling output, in order to study the general impact of activation of Rac1 on its site-specific ubiquitination. Moreover, K-R site-specific mutations were introduced into Q61L Rac1 to better distinguish the sole effect of Rac1 activation, rather than activating Rac1 with stimuli, which will in addition to Rac1 activate other targets as well. We agree that, if the K-R mutations were introduced into wild-type Rac1 instead of Q61L Rac1, a stimulation of Rac1 would provide more insight. Testing of the effects of different endogenous stimuli (e.g. S1P, Thrombin, VEGF, etc) is definitively of interest, but is beyond the scope of the present study.*

3. PAK-CRIB assays can have high variation, and it is best to include positive and negative controls to give some insight to min and max values – typically by adding excess GDP or GTPγS to the sample to prevent or enhance Rac1-PAK interactions.

*In our other experiments, we have included the T19N and Q61L Rac1 as negative and positive controls, respectively, and obtained the expected results. Furthermore, we perform the CRIB pulldown from cells, where an excess of GTP over GDP is present. Although addition of GDP or GTPγS is relevant for cell-free assays, ectopic addition of GDP or GTPγS to our cells will most likely not resolve the biological variability. Finally, the high variation in Rac1 activation assays is mostly observed using the GST- PAK CRIB domain, isolated from bacteria. Here, we use a, well established, shorter CRIB peptide linked to a biotin rather than a GST tag, which is chemically synthesized and results in much less technical variation in these experiments.*

4. Do the inhibitor treatments in Fig. 1 also enhance/prevent CRIB pulldown of Cdc42? Do inhibitors have an effect on PAK autophosphorylation as a marker of downstream Rac1-GTP activity, rather than just an interaction with recombinant CRIB domain?

*We analyzed activation of Cdc42 in our assays, following the same treatment as we have used for analysis of Rac1, but were unable to conclude that Cdc42 activity (which is low and hard to detect in our EC) is increased by any of the compounds tested. This result suggests that the effect of MLN7243 is specific for regulation of Rac1 activation in cells.*

*In addition, we analyzed pPAK levels under the conditions of Fig,1, but could not detect a significant increase in signal. However, analysis of pPAK in our hands is notoriously difficult and certainly not as sensitive as the CRIB-peptide pull down. The timing of optimal pPAK detection could also be not optimal in this experiment, as this was not designed to also analyse further downstream (and mostly transient) events. In combination with our reply to question 1, we feel that we are not altering directly the Rac1-CRIB domain interaction.*

5. In Fig. 2D, it is not clear if site-specific mutants of Rac1 that affect Rac1 activity are altering Rac1 ubiquitinylation – or, if Rac1 activity itself globally regulates ubiquitinylation. Did the authors measure ubiquitinylation of any other specific proteins or total cell lysates in these samples? For instance, the Q61L lane seems to have more background signal in general. Overall, it is very concerning that the authors never examine total protein ubiquitinylation in their samples (anti-ub Western blot), as the results could just be an effect of protein overexpression on homeostasis.

*Following this suggestion of this reviewer, we have performed an additional control experiment, shown in the figure below. This experiment was performed in HEK293T cells, and we have included untransfected HEK293T cells treated with DMSO, MLN7243 and PR619 for two hours as controls. Western blot was performed using the FK-2 antibody, detecting the total ubiquitinated proteins (not free ubiquitin), Rac1 antibody and GAPDH antibody. As can be seen, MLN7243 completely abrogates protein ubiquitination. Total ubiquitination was not affected in the other samples. PR619 treatment did not cause a significant decrease in ubquitination pattern, possibly due to smaller contribution of the deubiquitination process to the total ubiquitination pattern and too short treatment duration. Furthermore, we have not observed differences in the overall ubiquitination pattern between untransfected cells and cells overexpressing Rac1, regardless of its activation status. Finally, we also did not observe any differences in total ubiquitination patterns between the WT and Q61L Rac1 overexpressing cells with either myc or mCherry tag.*

6. How many different RhoGDIs are expressed by HEK293T cells and which one is examined in Fig. 3? The authors are likely following RhoGDIα/RhoGDI1/ARHGDIA – but RhoGDI2/ARHGDIB may be more relevant to Rac1 interactions and localization.

*The antibody we have used to detect RhoGDI in our assays is not specifically raised against only one isoform of RHoGDI, therefore we assume that all isoforms are detected. We expect that RhoGDI1 is mainly expressed in these HEK293T cells. Generally, RhoGDI1 is ubiquitously expressed, whereas RhoGDI2 is preferentially expressed in hematopoietic cells and RhoGDI3 is expressed in the brain, testes and pancreas (1). It is possible that RhoGDI2 is more relevant in hematopoietic stem cells where it might regulate the activity of Rac2, but it is more likely that RhoGDI1 is the most relevant one for Rac1 regulation in endothelial cells and other cell types (2).*

7. A main conclusion of the manuscript appears to be “that the lack of K16 ubiquitination is primarily due to a loss of its GTP-binding capacity”. However, the data supporting this conclusion are not very convincing. It seems more likely that mutations (even K->R) within the Rac1 GTP binding domain severely alter something about Rac1 structure/function in a manner limiting any function around Rac1. The authors do provide some control information here in Supp. Fig. 2 demonstrating that K16R on its own does not affect Rac1-CRIB interactions, but it is difficult to distinguish “zero” levels from background without an mCherry alone or even blank pulldown control.

*We agree with the reviewer on the notion that mutations in the Rac1 GTP binding domain severely alter the activity of Rac1. We included the new Supplemental Figure 4 with the model of K16R mutant of Rac1, where we show that this mutation indeed might interfere with the GTP binding rather than with ubiquitination of the Rac1. We have shown in Figure 4 by CRIB pulldown that Rac1 Q61L, with high levels in the pulldown fraction, loses its activity when the K16R mutation is introduced. The activity level of Rac1 Q61L K16R hereby is similar to that of Rac1 WT, which is significantly lower and shown in the same experiment. Furthermore, in some of our CRIB pulldown assays we had empty vector control (e.g. Figure 5D), where we have never observed any difference in CRIB binding between cells transfected with empty vector or WTRac1. Therefore, in other assays we rather compared the binding between Rac1 WT and activating mutants of Rac1, than to compare the “zero” background from mock-transfected or cells transfected with constructs expression the tag only. Also in the Supplemental Figure 3, we have compared the CRIB binding efficiency of the WT Rac1 and Rac1 V12 mutant. Here we show that Rac1 constructs with only KR mutations did not significantly differ from WT Rac1 in their CRIB binding capacity, but were all much less potent in that respect in comparison to the activating V12 Rac1 mutant.*

8. Controls for uniform transfection and consistent levels of exogenous protein expression are not clear.

*We respectfully disagree with this comment of the reviewer 1. In all our blots we have used GAPDH (Figures 1a, 2c, 2d, 3a, 5c, 5d, 5e, Suppl. figures 1 and 3) which is a housekeeping gene, as loading control for the amount of cell lysate that was loaded. In addition, the mCherry (Figures 2c, 2d, 3a and Suppl. figure 3) or myc (Figures 5c, 5d and 5e) antibodies in the ‘input’ blots of our CRIB pulldowns, ubiquitination assays or COIPs show equal levels of transfection in all analyzed samples.*

Minor

1.The Abstract includes too much background information, is too vague and should include more specific summaries of Results and Conclusions.

*Following the suggestion of this reviewer we have adjusted the abstract.*

2. In general, many parts of the manuscript are too wordy and focused on Background information. There is no need to repeat Background details again and again in Abstract, Intro, Results, etc. There is so much editorial language in the Results section, that it is difficult to

*Following the suggestion of this reviewer we have adjusted small parts in the results section to better clarify our statements.*

3. The use of the term “in vivo” in reference to Fig. 2 is not ideal - suggests an effect in an animal model or human subject.

*We have changed this reference to “in cells”.*

4. Fig. 3 is informative – but perhaps would be best kept as a Supplementary figure confirming previous results – or as part of a larger figure. For instance, it might make the presentation of the data better if the authors aligned the graph in Fig. 3B above similar graphs in Fig. 4BC to show how GDI interactions and Rac1 activity relate.

*We thank the reviewer for their suggestion. However, we feel that separating Figure 3 from Figure 4 enables more clear description of the obtained results on GDI interaction with Rac1 activating mutants in HEK293T cells. Figure 4, which is displaying cellular effects of Rac1 activating mutants in HUVECs, is more compact and easier to follow without addition of results on GDI interaction in another cell type in the middle of the figure.*

5. Fig. 5B is very helpful. It would be nice to have a related (perhaps Supplemental) panel (or reference citation) aligning Rac1 protein sequences from multiple species to show lysine conservation.

*We thank the reviewer for their suggestion and have included Rac1 alignment of multiple species as Supplemental Figure 2.*

6. Is there a reason for difference in Rac1 staining of KR mutants in Fig. 6? This appears to be independent of effects on Rac1 activity and could be of interest.

*We agree that differential staining of Rac1 was observed, however this was not a consistent observation and we therefore did not draw any conclusions on this aspect.*

Reviewer #2:

In the manuscript entitled “The interplay of Rac1 activity, ubiquitination and GDI binding and its consequences for endothelial cell spreading”, Majolee et al investigate the relationship between Rac1 activity, ubiquitination and binding to RhoGDI using a range of Rac1 mutants including activating, inhibitory or fast-cycling mutations. The authors positively correlate the activity and ubiquitination of the mcherry-Rac1 mutants They also assess endothelial cell spreading upon expression of the various mutants and positively correlate the solidity of EC shape (cell spreading) with Rac1 activity (as measured by CRIB pull-down assay in all cases). They next set out to assess the effect of ubiquitination on Rac1 activity and RhoGDI binding by individually mutating a number of lysine residues in a Q61L Rac1 mutant background. The K16R mutation blocks both activation and ubiquitination of Rac1.

Overall, this manuscript is interesting as the authors set out to investigate the relationship between Rac1 activity, ubiquitination and RhoGDI binding. It is clearly written and scientifically sound regarding the experiments carried out and the conclusions drawn by the authors. I think the authors satisfactorily correlate endothelial cell spreading with Rac1 activity, so I won’t comment these experiments any further. However, I think the conclusions that can be drawn from this set of experiments and the underlying strategy are somehow limited because it is difficult, solely based on the experiments provided, to determine which effects are dependent on the ubiquitination and on the mutations on Rac1 structure. Therefore, I wouldn’t recommend it for publication unless additional experiments would support and back up the authors conclusions. It is definitely conceptually appealing but falls short of addressing a critical issue under this form in my opinion.

Major points:

My understanding of the regulation of Rac1 by ubiquitination and degradation was that this was a mean to terminate Rac1 activation in case of sustained or irreversible Rac1 activation. For instance, following CNF1 treatment, Rac1 gets irreversibly modified and constitutively activated which triggers its proteasomal degradation. Here, I am struggling to understand the authors conclusions and how they include ubiquitination in the GTPase cycle.

*We thank the reviewer for their positive comments. Regarding this question; it is clear that the CNF-induced constitutive activation of Rac1 is an unusual means of long term activation. However, these studies did serve to link Rac1 activation to its ubiquitination, as also seen with the Q61 variant as well, by us and others. That Rac1 ubiquitination, linked to its activation, also happens with endogenous Rac1 can be deduced from other recent studies (3-6). Here, the authors identified E3 ligases responsible for ubiquitination of (endogenous) active Rac1 (XIAP/cIAP, HACE1, TRAF6, etc).*

*As an example, a recent study (Turgu, Oncogene, 2021) showed that the E3 ligase for Rac1 , HACE1, is upregulated by hypoxia and reduces HIF1a expression by targeting active Rac1 for degradation. Thus, ubiquitination of endogenous active Rac1 is a bona fide means to limit Rac1 signaling. However, it is unknown why cells have at least two alternatives to inactive Rac1 : through GAP-mediated increased nucleotide hydrolysis and through ubiquitination. This topic is subject of ongoing studies in our lab.*

In the first part of the manuscript, they assess the correlation between activity, ubiquitination and GDI binding. I think it is fair to conclude from their experiments that activity and ubiquitination and dissociated from GDI binding with the mutants evaluated here, which incidentally does not necessarily mean that endogenous Rac1 behaves similarly. I think it would be worth noting that accordingly to the model discussed above, non-cycling active mutants (Q61L, G12V) are more ubiquitinated than activated fast-cycling mutants (P29S, F28L).

*We thank the reviewer on their comment and agree with this notion. We have adapted our discussion accordingly (page 15, line 378-381).*

One major criticism here that is not addressed or mentioned by the authors would be that they use mcherry-Rac1 constructs. This construct is most likely properly prenylated, but the potential caveat is that mCherry creates a massive steric hindrance on Rac1 that may affect its behavior (as compared to endogenous Rac1 or myc-tagged Rac1 used afterwards). This has been mentioned by others and justified the use of alternative tagging strategies in other studies (Golding et al., 2019). This seems quite critical to me to at least address this point since the focus of this manuscript is to assess the cycling of Rac1.

*A number of previous studies have used FRET sensors to localize Rac1 activity. These sensors are much bigger than mCherry-Rac1 and still show expected activation and localization differences upon stimulation (7, 8).*

*Both our myc- and mCherry-tagged Rac1 display the expected differences in CRIB pulldown assays (active vs. dominant negative Rac1 mutants) and in the induction of morphological changes in HUVECs, therefore we conclude that mCherry- or myc-tagged Rac1 do recapitulate endogenous Rac1 behavior to a large extent as also reported in literature previously (e.g. Michaelson et al. 2001 (2)).*

*Following the suggestion of this reviewer, we have included a sentence, related to this point, in the discussion (page 14, lines 360-362).*

I am more skeptical with the second half of the manuscript. The authors set out to investigate the effect of ubiquitination on the activity of Rac1 by using the classical active non-cycling Q61L mutant and individually mutating a number of selected Lysine residues. As previously, the authors compare activation, ubiquitination and RhoGDI binding. The Q61L K16R mutant stands out by its reduced activity which correlates with its reduced ubiquitination. This is where I am mostly conceptually puzzled:

Regarding ubiquitination, it looks like mutating this single residue completely blocks any ubiquitination of Rac1, does that mean this is indeed the only ubiquitinated residue on Rac1 or that it primes Rac1 for further ubiquitination on other Lysines?

*Lysine 16 seems to be more important for the GTP binding than for ubiquitination process itself. Although Li et al in their work from 2017 suggest that lysine 16 is required for Traf6-mediated Rac1 ubiquitination, the authors never showed the effect of K16R mutation on Rac1 ubiquitination with purified proteins. This work was for us reason to look at the lysine 16 although this particular lysine is buried in the Rac1 structure and is likely not accessible for the interaction with E3 ligase. More careful analysis of the Rac1 structure and its GTP binding pocket by structure modelling shows that K16 is required for GTP binding (now Supplemental Figure 4 a and b). Mutation to arginine disrupts the interaction with GTP, since the hydrogen bond between the arginine side chain and the second phosphate group of GTP is much weaker due to large distance of 3.44* *Angstrom compared to 2.65 Angstrom between K16 side chain and second phosphate group of GTP. This leads most likely to an inability of K16R Rac1 to efficiently bind GTP. Since the K16 is therefore key to Rac1 being activated, the residue is also essential for Rac1 to be ubiquitinated. The data in Fig 5 C show that K147 is essential for ubiquitination: in this construct K16 is intact but is apparently not ubiquitinated. Thus, we conclude that K16R indirectly, and K147R directly blocks ubiquitination of active Rac1.*

I am not sure to understand the mechanistic effect of this mutation. My understanding is that the Q61L mutation favors GTP loading and prevents any hydrolytic activity hence increased activity. Does it mean that the K16R mutation prevents GTP loading of Rac Q61. Therefore, this would mean that GTP loading would be ubiquitination-dependent which is quite controversial regarding what is known from CNF1 studies for instance.

*We agree on this notion, please see our previous response. We have adapted our results and discussion sections accordingly.*

Alternatively, as mentioned by the authors in the discussion, another explanation could simply be that this K16R mutation somehow disrupts Rac structure/function/ GTP binding… This could also hold true with any of the other Lysine mutants and makes it difficult to interpret the data and draw conclusions solely based on these experiments.

I fear that unless the authors provide additional data to establish that these mutants can be used safely, it is difficult to confidently conclude anything from those experiments.

*We chose the lysines to mutate based on known data from phosphosite.org, published literature and on their position in the Rac1 structure. While K16 clearly points inwards into the protein 3D structure and is localized in the GTP-binding pocket, the 3D structure of Rac1 shows that all other mutated lysine side chains point outward. Therefore, their mutation to positively charged arginines is unlikely to significantly interfere with the Rac1 structure. This is obvious also from the alignment of the WT Rac1 (cyan) and K147R structure (magenta) in the figure below.*

*As opposed to the K16R mutant, Rac1 Q61L K147R retains its activity and can induce the same morphological changes in HUVEC compared to Rac1 Q61L, while ubiquitination is completely abolished (see data in Figure 5C).*

Minor point

The introduction is clear and states the rationale for this study, but I had the feeling that a number of (appropriate) statements would benefit from citing the articles demonstrating the statement such as lines 38, 41, and 49 for instance.

*We thank the reviewer for their suggestion and have altered the introduction accordingly.*

**References**

1.	Garcia-Mata R, Boulter E, Burridge K. The 'invisible hand': regulation of RHO GTPases by RHOGDIs. Nat Rev Mol Cell Biol. 2011;12(8):493-504.

2.	Michaelson D, Silletti J, Murphy G, D'Eustachio P, Rush M, Philips MR. Differential localization of Rho GTPases in live cells: regulation by hypervariable regions and RhoGDI binding. J Cell Biol. 2001;152(1):111-26.

3.	Oberoi TK, Dogan T, Hocking JC, Scholz RP, Mooz J, Anderson CL, et al. IAPs regulate the plasticity of cell migration by directly targeting Rac1 for degradation. EMBO J. 2012;31(1):14-28.

4.	Torrino S, Visvikis O, Doye A, Boyer L, Stefani C, Munro P, et al. The E3 ubiquitin-ligase HACE1 catalyzes the ubiquitylation of active Rac1. Dev Cell. 2011;21(5):959-65.

5.	Andrio E, Lotte R, Hamaoui D, Cherfils J, Doye A, Daugaard M, et al. Identification of cancer-associated missense mutations in hace1 that impair cell growth control and Rac1 ubiquitylation. Sci Rep. 2017;7:44779.

6.	Li T, Qin JJ, Yang X, Ji YX, Guo F, Cheng WL, et al. The Ubiquitin E3 Ligase TRAF6 Exacerbates Ischemic Stroke by Ubiquitinating and Activating Rac1. J Neurosci. 2017;37(50):12123-40.

7.	Itoh T, Watabe A, Toh EA, Matsui Y. Complex formation with Ypt11p, a rab-type small GTPase, is essential to facilitate the function of Myo2p, a class V myosin, in mitochondrial distribution in Saccharomyces cerevisiae. Mol Cell Biol. 2002;22(22):7744-57.

8.	Reinhard NR, Mastop M, Yin T, Wu Y, Bosma EK, Gadella TWJ, Jr., et al. The balance between Galphai-Cdc42/Rac and Galpha12/13-RhoA pathways determines endothelial barrier regulation by sphingosine-1-phosphate. Mol Biol Cell. 2017;28(23):3371-82.
